# Searching the Staphylococcal Superantigens: Enterotoxins A, B, C, and TSST1 in Synovial Fluid of Cases With Negative Culture Inflammatory Arthritis

**DOI:** 10.5812/jjm.11647

**Published:** 2014-07-01

**Authors:** Mohammad Reza Shokrollahi, Samileh Noorbakhsh, Mohammad Aliakbari, Azardokht Tabatabaei

**Affiliations:** 1 Department of Medicine, Qom University of Medical Sciences and Health Services, Qom , IR Iran; 2 Research Center of Pediatric Infectious Diseases, Iran University of Medical Sciences, Tehran, IR Iran

**Keywords:** Arthritis, *Staphylococcus aureus*, Toxic Shock Syndrome Toxin-1

## Abstract

**Background:**

Accurate and rapid diagnosis of bacterial arthritis is not always possible in unvaccinated (*Streptococcus pneumoniae* and *Haemophilus influenzae* type B) children in Iran.

**Objectives:**

Searching the staphylococcal superantigen (entrotoxin A, B, C and TSST1) in synovial fluid of cases with inflammatory arthritis.

**Patients and Methods:**

This cross sectional study was implemented in the pediatric and orthopedic wards, Rasoul Akram Hospital, Tehran, Iran (2008-2010) upon synovial fluid (SF) aspirated from 66 children (five months to 16 years; mean age 11 ± 3.8 years) with monoarthritis. Staphylococcal supperantigens (enterotoxins A, B, C, TSST1) were assessed by Enzyme-linked immunosorbent assay (ELISA) in synovial fluid of cases with inflammatory arthitis. Staphylococcal superantigens compared between cases with positive and negative *Staphylococcus aureus* culture (P < 0.05 was significant)

**Results:**

*S. aureus* was the most common cause of septic arthritis. Positive *S. aureus* culture in SF was reported in 10.6% (7/66) of the cases. Enterotoxin A was the least common type of superantigens found even in SF negative culture; 47% of the cases had one or more staphylococcal superantigens. Enterotoxin A was the least common type in SF; there was poor agreement between positive culture for *S. aureus* and presence of enterotoxins B, C, and TSST1 in SF, and intermediate agreement (KAPPA Index = 0.67) for enterotoxin A.

**Conclusions:**

A possible role (%47) for staphylococcal toxins was defined even in SF negative cultures obtained from monoarthritis cases. Failure in isolation of organisms might be due to natural un-growth of microorganism in synovial fluid, and previous antibiotic usage or low technical methods. It could not be determined from the data obtained in the current investigation whether or not staphylococcal toxins (superantigens) play a pathogenic role without direct invasion of the organism. It is recommend to use complementary methods for searching the *S. aureus* superantigens in future studies.

## 1. Background

Acute complications and risk of long-term morbidities in septic arthritis are common in infants and older children (1). Signs and symptoms are often nonspecific and it is not always possible to make a differential diagnosis between bacterial and aseptic arthritis. Synovial fluid (SF) cultures which are positive in 80% of arthritis cases are the gold-standard test for diagnosis (2). Protein and glucose level or white blood cell (WBC) count in SF were not specific and sensitive enough for diagnosis. Gram-staining of SF (positive in 50% to 80% of cases) is an insensitive technique and must be confirmed by culture (2). Li et al. reported the elevated factors such as WBC (> 11,000 cells/mm), ESR ( > 30 mm/hr), or SFA-WBC (> 50,000 cells/mm) in 73 adults who had septic arthritis confirmed by positive arthrocentesis culture or operative findings as 48%, 96%, and 64%, respectively (3, 4). 

Except DNA-Polymerase Chain Reaction (DNA-PCR) method, other laboratory tests do not rule out septic arthritis with accuracy. Recently, various biological markers like C-Reactive protein (CRP), procalcitonin, or STREM-1 have been useful to diagnose and differentiate between bacterial and non-bacterial infections (5, 6). The Centers for Disease Control and Prevention estimate that 1.3 million hospital patients develop *Staphylococcus aureus* infections every year, prolonging hospital stays and increasing treatment costs. In fact, food poisoning affects 60 to 80 million people worldwide each year and results in approximately six to eight million deaths.

Bacterial superantigens (for example, enterotoxins) are microbial proteins (23 to 30 kDa) that unlike conventional antigens, bypass antigen processing by binding to a major histocompatibility class II (MHC II), and bridge MHC II with the T cell receptor and T cell activation .These potent virulence factors stimulate several immunological processes and play a role in the maintenance of inflammation. A close association of *S. aureus* with several inflammatory diseases has been investigated with or without systemic involvement. The bacterial superantigen secreted by *Staphylococcaceae* family has been the subject of intense research over the last decade. Staphylococcal superantigens directly stimulate a massive inflammatory response which has some roles in the pathogenesis of acute diseases such as food poisoning, toxic shock Kawassakii, and some types of chronic inflammatory diseases described in recent years.

*S. aureus* is one of the common causes of bone and joint infections in humans. *S. aureus* can infect osteoblasts and survive intracellularly. Eliminating these pathogens is difficult for osteoblasts. Three to six superantigens produced by *S. aureus* are able to stimulate up to 20% of the T-cell population in a non-specific way. The enterotoxins produced by *S. aureus* will be emphasized since this pathogen is an emerging health concern in the environment, and hospital infections (7). These lethal toxins trigger an excessive cellular immune response leading to toxic shock syndrome in childhood (8, 9). Chemokines are small, chemoattractant cytokines which play key roles in the accumulation of inflammatory cells at the site of inflammation. Chemokines have an important role in the joints of patient with arthritis (10). 

Recently, some authors have described osteoblasts to secrete pro-inflammatory cytokine; IL-12. The central role of IL-12 for initiation of Th1-type, cell-mediated, and immune responses is described. Real-Time PCR (RT-PCR) and immunofluorescent techniques were used to determine the IL-12 induction in human and mouse osteoblasts (11, 12). Schutyser et al. reported that the CCL18/PARC protein was spontaneously secreted by PBMC induced by staphylococcal enterotoxins (A, B) and IL-4 (11). Arad et al. reported therapeutic effects of superantigen antagonist against shock (12). Many Iranian authors reported the clinical and etiology of arthritis (13, 14). Bacterial arthritis continues to be the most important illness with high morbidity among unvaccinated (*Streptococcus pneumoniae* and *Haemophilus influenzae* type B) children in Iran (13). Biologic markers were used to differentiate the bacterial from aseptic arthritis in Iran (15, 16). Talebi-Taher et al. studied the ability of serum inflammatory markers to discriminate between septic versus inflammatory arthritis (16).

## 2. Objectives

Accurate and rapid diagnosis of bacterial arthritis is essential for earlier treatment and obtaining a good outcome in children. Definite and rapid discrimination of pyogenic arthritis from aseptic arthitis is very important in children. It would be used effectively to shorten unnecessary empiric antibiotic therapy in non-bacterial arthritis. In present study we searched the staphylococcal superantigen (entrotoxin A, B, C and TSST1) in synovial fluid of cases with inflammatory arthritis. 

## 3. Patients and Methods

This cross sectional study was implemented in pediatric and orthopedic wards of Rasoul Akram Hospital in Tehran, Iran (2008-2010). This study was approved by the Ethical Committee in the Research Center of Pediatric Infectious Diseases in Tehran university of Medical Sciences (Code number: MT 207), and was performed according to the Declaration of Helsinki Principles. All of the participants were fully informed about the study and they signed the informed consent forms. Seventy seven synovial fluids samples were aspirated from children with monoarthritis and studied. Patients were classified as septic and aseptic arthritis.

### 3.1. Data Collection

Initially, a questionnaire was filled out by an authorized physician for each case (for example, age, gender, analysis of SF samples, biochemical parameters, Gram-stain, and SF culture in both convention and BACTEC medium). Total WBC and differential Gram-stain was performed on the SF centrifuged samples. The deposits were cultured on sheep blood agar (conventional culture), stored in candle jar (37°C) for 48 hours. The BACTEC Ped Plus medium (Becton Dickenson company) and automated system were used (Bio Merieux, France) for samples with negative conventional cultures. All the isolates were identified using standard techniques. Septic arthritis defined as inflammatory arthritis + positive SF culture or positive cultures of the blood + /-SF culture; positive direct Gram-stain of SF.

### 3.2. Aseptic Arthritis

Inflammatory arthritis + negative culture for bacteria on blood and SF culture/ negative direct Gram-stain of SF.

### 3.3. Exclusion Criteria

In the first step, all cases with poly arthritis such as migratory poly arthritis, rheumatics fever, immune deficiencies state, Brucellosis, TB, hemarthrosis, and etc. were excluded. In the second step, 11cases with septic arthritis (positive culture for other microorganisms except *S. aureus* such as *S. pneumonia*: five, *H. influenza*: two, Klebsiella spp.: one, *Neisseria meningitidis*: one; *Pseudomonas aureogenosa*: one; *Candida* *albicans*: one) were excluded.

### 3.4. Case Definition

Positive *S. aureus* culture (SF) reported in 10.6% (7/66) of the 66 selected cases, and seven cases with positive *S. aureus* culture (7/66) were diagnosed and compared with the 59 cases with negative culture. Superantigens were compared between the two groups. Staphylococcal superantigens enterotoxins A, B, C, and TSST1 were searched in synovial fluid by ELISA methods (enterotoxins A, B, C, and TSST1 ELISA kit; ABcam Company; USA).

### 3.5. Statistical Analysis

The SPSS software version 11.5 was employed for data analysis. The Student’s t- test was used to determine significant differences of means for all continuous variables. Chi-square values (CI 95%) were calculated for all categories at 0.05 level of significant. The likelihood ratio test to test for linear trend and interaction between variables was conducted. McNemar and computing kappa statistics were used for comparing the variables.

## 4. Results

In the 66 samples, 53.4% of the cases were male, and 46.6% female. Range of age was from five months to 16 years, mean age: 11±3.8 years. *S. aureus* (10.6%; 7/66) was the most common cause of septic arthritis. One or more types of staphylococcal superantigens were detected in 47% of the studied cases. Type of staphylococcal superantigens in synovial fluid of the cases is shown in Table 1. Enterotoxin A was the least common ones.

Positive culture for *S. aureus* in SF had poor agreement with the presence of TSST1 and enterotoxins B, C (KAPPA Index = 0.37 for TSST; KAPPA Index = −0.15 for SETB; KAPPA Index = 0.35 for SETC respectively) except for enterotoxin A which had an intermediate agreement (KAPPA Index = 0.67) 

**Table 1.** Type of Staphylococcal Superantigens in Synovial Fluid of the Cases ^a^

Type of staphylococcal Superantigens in Synovial Fluid	Enterotoxin A	Enterotoxin B	Enterotoxin C	TSST1
**Positive, No. (%)**	22 (39)	10 (18)	21 (39)	28 (47)
**Negative, No. (%)**	33 (61)	46 (82)	33 (61)	32 (56)
**Total**	56	56	54	60

^a^ Abbreviation: TSST, Toxic Shock Syndrome Toxin.

## 5. Discussion

The Gold-standard test to diagnose septic arthritis is SF culture. Many authors reported 80% positive SF cultures in the best methods (1, 3). Li et al. showed that routine laboratory tests were useful to confirm a suspected diagnosis, to assess disease activity, and to measure the response and toxicity of the treatment (3). Gram-staining of SF revealed bacteria in about 50% to 80% of the cases but was an insensitive technique and must be confirmed by culture. SF leukocyte count and concentration of protein and glucose lack specificity and sensitivity for diagnosis (1). The sensitivity of PMN predominance for aseptic arthritis was 57% whereas the specificity was 10%. The positive predictive value for PMN predominance in aseptic disease was 81% but the negative predictive value was 31% (1). 

In the setting of an acute arthritis in the present study 11 (n = 77) proved septic arthritis caused by organisms except *S. aureus* (for example, *S. pneumonia*: five, *H. influenzae*: two, *Klebsiella* spp.: one, *N. meingitis*: one, *P. aeruginosa*: one, *C. albicans*: one) were found and excluded. *S. aureus* was the most common type of organisms isolated in SF (7/18) of septic arthritis in childhood. Wang et al. reported *S. aureus* as the predominant causative microorganism in 43% (n = 58) of the septic arthritis (mean age = 3 years) which is very close to that of the current study (2). The low rate of microorganism detection in the studied cases might be due to natural un-growth of microorganism in synovial fluid. Previous antibiotic treatment might explain the false negative cultures or low technical methods in some cases. 

Bacterial arthritis diagnosed in the current study cases was far from previous studies in Iran reported in adult cases (45%); and 70% in the pediatric population (13, 14). The older age of the cases (mean age: 11 ± 3.8 years) in the present study or undefined inclusion criteria for selection of cases could explain these differences (14). Relatively low number of laboratory confirmed cases of bacterial arthritis observed in the present study, in cases with strong clinical suspicion of infection or cases with inflammatory arthritis, and signs and symptoms of bacterial arthritis in infants and older children are often nonspecific and it is not always possible to make a differential diagnosis between bacterial and aseptic arthritis (1). Reactive arthritis is more frequent than bacterial arthritis in childhood (1-3). Some of the cases with negative culture, and negative Gram-stain samples are categorized as inflammatory non-septic or reactive arthritis (3). Li et al. concluded that laboratory tests do not rule out septic arthritis with accuracy in 73 adult cases with septic arthritis (3). Using the confirmatory diagnostic tests (PCR) was unavailable in the present study to rule out the false negative cultures (4-6). Here, *S. aureus* was the most common cause of septic arthritis in the studied children which was very close to all previous Iranian studies and also those of Li et al. and Wang et al. in Taiwan(1-3, 13, 14).

In contrast to culture, *S. aureus* toxins were detected in synovial fluid of 47% of the arthritis cases. Staphylococcal superantigens have been implicated in the pathogenesis of some inflammatory diseases (7). No correlation was observed between isolation of *S. aureus* from SF and determination of *S. aureus* superantigens. Staphylococcal superantigens (TSST, enterotoxins A, B, and C) might have a prominent role in children arthritis. *S. aureus* toxins might have a role in inducing the arthritis determined from the data obtained in the current investigation.* S. aureus* is the most common cause of nosocomial and community acquired infection in children. The risk of emergence of methicillin-resistant strains causing community-acquired infections is widespread worldwide. 

Skin and soft tissue infections as well as bone and joint involvement are frequent in children. Superantigens which are produced by Gram-positive and negative bacteria are microbial proteins that are able to stimulate T-cell population in a non-specific way. Superantigens had the potency to induce inflammation by extensive cytokine release after T-cell stimulation, T-cell-mediated cytotoxicity, and finally tissue damage. MHC class II molecules serve as superantigen receptors. Superantigens have a high affinity for binding to the alfa-chain of the MHC class II molecule, whereas the carboxy-terminal site is responsible for a strong binding to the T cell receptor (TCR).* S. aureus* secretes toxins leading to specific diseases: enterotoxins cause food-poisoning and exofoliatines causes generalized exfoliation and bullous impetigo. 

Staphylococcal toxins have the capacity to act as superantigens, bypassing normal antigen processing, provoking polyclonal activation of a large number of T cells, and the release of cytokines. It has been shown that superantigens stimulate B-cells to increase production of allergen-specific IgE. Staphylococcal toxic shock syndrome is caused by a group of superantigens that includes toxic shock syndrome toxin-1 (TSST-1) and staphylococcal enterotoxins A, B, and C (7, 8). Floret et al. discussed the clinical manifestations of streptococcal and staphylococcal toxinic diseases (7). This polyclonal activation has been observed in some pediatric diseases of unknown origin, it has strong evidence in Kawasaki syndrome, probable effect in sudden death syndrome in infants and in acute exacerbations of atopic eczema and psoriasis. TSST-1 is a superantigen which can also be immunogenic. In patients with atopic dermatitis neutralizing anti-TSST-1-IgG antibodies anti-toxin-IgE antibodies are detectable. These toxins, released by some *S. aureus* strains, are a cause of the toxic shock syndrome (TSS). The disease may develop during any staphylococcal infection like a super infection of a skin disease. 

Recent studies determined the involvement of *S. aureus* enterotoxins in lower airway diseases such as severe asthma and exacerbated chronic lung diseases, probable exposure to staphylococcal exotoxins in the blood and polyp tissue of patients with chronic rhinosinusitis with nasal polyposis (7-10). Superantigens produced by *S. aureus* are one of the most lethal toxins 7, therefore it is very important to have easier and quicker tests to determine the toxin production pattern of *S. aureus*. Some conventional methods such as ELISA, immunodiffusion or agglutination test to detect *S. aureus* toxins are available now. Indeed, the new sensitive and specific method (PCR) is introduced by some authors. Sensitivity and specificity of the PCR test has been reported from 82% to 100%. Recently, a multiplex-PCR test has been reported, which is able to detect the toxin-producing capacity of staphylococcal strains rapidly (8-12). Arad et al. and Kaempfer et al. studies defined the antagonist activity of this peptide, thus identifies a novel domain in superantigens that is critical for their toxic action (9-12).

Iwamoto et al. reported the effects of chemokines in the joints of rheumatoid arthritis patients (10). Schutyser et al. found that monocytes respond to Gram-positive bacterial infections by producing CCL18/PARC in the synovial cavity (11). Here, a possible role for staphylococcal toxins (superantigens) was defined in arthritis cases without isolation of organisms from synovial fluid. Unlike conventional antigens, staphylococcal toxins can bypass antigen processing by binding to a specific groove outside the usual peptide-binding site of MHC II which are able to bridge MHC II with the (TCR) unleashing robust T cell activation. Whether or not staphylococcal toxins (superantigens) play a pathogenic role without direct invasion of the microorganism needs future larger studies on the *S. aureus* superantigens. The current study agrees with Li et al. (3) and Wang et al. (2) that the confirmatory diagnostic test (PCR) would be helpful to decrease the false negative cultures with strong clinical suspicion of infection or other inflammatory conditions. 

Due to failure in isolation of bacteria, the current study preferred to use complementary methods in children. These results would be valuable information as there is no similar study looking at the staphylococcal toxins in SF of children with arthritis. All data based on the assumption criteria in patients have been correctly categorized to the culture or Gram-stain. These data are critical as all further roles for staphylococcal toxins (superantigens) in children with arthritis (and negative SF culture). The main limitation of the study was the small study population, especially in younger age (< 2 years). This is further complicated by the relatively low number of laboratory confirmed cases of bacterial arthritis in the study. Confirmatory diagnostic test (PCR) to decrease the false negative cultures was not used.

### 5.1. Ethics Consideration

Ethical Committee in the Research Center of Pediatric Infectious Diseases (affiliated to Tehran University of Medical Sciences) reviewed and approved the waiver of authorization to use the Protected Health Information (PHI) for research purposes of the following study (Ethics code number: MT 207). The following requested PHI was necessary to conduct the study. Insertion of the patient information to be used or disclosed, or attach documentation of the information. The use or disclosure of PHI involves no more than minimal risk. Granting the waiver will not adversely affect privacy rights and welfare of the individuals whose records will be used. The project could not be practicably conducted without a waiver and use of PHI. The privacy risks are reasonable relative to the anticipated benefits of the research. An adequate plan to protect identifiers from improper use and disclosure is included in the research proposal. An adequate plan to destroy the identifiers at the earliest opportunity, or justification for retaining identifiers, is included in the research proposal. The project plan includes written assurances that PHI will not be re-used or disclosed for other purposes. Whenever appropriate, the subjects will be provided with additional pertinent information after participation.
